# Crystal structure, Hirshfeld surface analysis, inter­action energy and energy framework calculations, as well as density functional theory (DFT) com­putation, of methyl 2-oxo-1-(prop-2-yn­yl)-1,2-di­hydro­quinoline-4-carboxyl­ate

**DOI:** 10.1107/S2056989023007557

**Published:** 2023-09-08

**Authors:** Ayoub El-Mrabet, Amal Haoudi, Samira Dalbouha, Mohamed Khalid Skalli, Tuncer Hökelek, Frederic Capet, Youssef Kandri Rodi, Ahmed Mazzah, Nada Kheira Sebbar

**Affiliations:** a Laboratory of Applied Organic Chemistry, Faculty of Science and Technology, University of Sidi Mohamed Ben Abdellah, BP 2202, Fez, Morocco; b Laboratory of Organic Chemistry and Physical Chemistry, Research Team: Molecular Modeling, Materials and Environment, Department of Chemistry, Faculty of Sciences, University Ibn Zohr in Agadir, BP 8106 Agadir, Morocco; c Laboratory of Spectroscopy, Molecular Modeling, Materials, Nanomaterials, Water and Environment, CERNE2D, Faculty of Sciences, Mohammed V University in Rabat, Av. Ibn Battouta, BP 1014, Rabat, Morocco; d Department of Physics, Hacettepe University, 06800 Beytepe, Ankara, Türkiye; e University of Lille, CNRS, UMR 8181, UCCS, Unité de catalyse et Chimie du solide, F-59000 Lille, France; f University of Lille, CNRS, UAR 3290, MSAP, Miniaturization for Synthesis, Analysis and Proteomics, F-59000 Lille, France; g Laboratoire de Chimie Bioorganique Appliquée, Faculté des Sciences, Université Ibnou Zohr, Agadir, Morocco; Vienna University of Technology, Austria

**Keywords:** crystal structure, π-stacking, C—H⋯O hydro­gen bonds, di­hydro­quinoline

## Abstract

In the title mol­ecule, C_14_H_11_NO_3_, the di­hydro­quinoline core deviates slightly from planarity, indicated by the dihedral angle of 1.07 (3)° between the two six-membered rings. In the crystal, layers of mol­ecules almost parallel to the *bc* plane are formed by C—H⋯O hydro­gen bonds. These are joined by π–π stacking inter­actions. A Hirshfeld surface analysis revealed that the most important contributions to the crystal packing are from H⋯H (36.0%), H⋯C/C⋯H (28.9%) and H⋯O/O⋯H (23.5%) inter­actions. The evaluation of the electrostatic, dispersion and total energy frameworks indicates that the stabilization is dominated by the dispersion energy contribution. Moreover, the mol­ecular structure optimized by density functional theory (DFT) at the B3LYP/6-311G(d,p) level is com­pared with the experimentally determined mol­ecular structure in the solid state. The HOMO–LUMO behaviour was elucidated to determine the energy gap.

## Abstract 2

In the crystal of the title com­pound, C—H⋯O hydro­gen bonds link the mol­ecules, enclosing 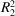 (10) and 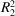 (16) ring motifs, into layers almost parallel to the *bc* plane. The layers are further connected by π–π stacking inter­actions.

## 1. Chemical context

Quinoline derivatives form a class of heterocyclic com­pounds that have received much attention due to their biological and pharmacological activities (Filali Baba *et al.*, 2019 ▸; Hayani *et al.*, 2021 ▸). They are used in the pharmaceutical industry because of their anti­microbial (Katoh *et al.*, 2004 ▸; Abdel-Wahab *et al.*, 2012 ▸), anti-inflammatory (Leatham *et al.*, 1983 ▸), anti­hypertensive (Muruganantham *et al.*, 2004 ▸), anti­biotic (Mahamoud *et al.*, 2006 ▸), anti-HIV (Wilson *et al.*, 1992 ▸; Strekowski *et al.*, 1991 ▸) and corrosion inhibitive activities (Filali Baba *et al.*, 2016*a* ▸,*b* ▸). They are also considered as an important scaffold for the development of new pharmaceutically active agents (Filali Baba *et al.*, 2020 ▸; Bouzian *et al.*, 2018 ▸).

In continuation of our research work devoted to the study of O-alkyl­ation and N-alkyl­ation reactions involving quinoline derivatives, we report herein the synthesis and the mol­ecular and crystal structures of methyl 2-oxo-1-(prop-2-yn­yl)-1,2-di­hydro­quinoline-4-carboxyl­ate, obtained by an alkyl­ation reaction of methyl 2-oxo-1,2-di­hydro­quinoline-4-carboxyl­ate using an excess of propargyl bromide as an alkyl­ating reagent in phase transfer catalysis (PTC). Moreover, a Hirshfeld surface analysis and inter­action energy and energy framework calculations were performed. The mol­ecular structure optimized by density functional theory (DFT) at the B3LYP/6-311G(d,p) level is com­pared with the experimentally determined mol­ecular structure in the solid state. 
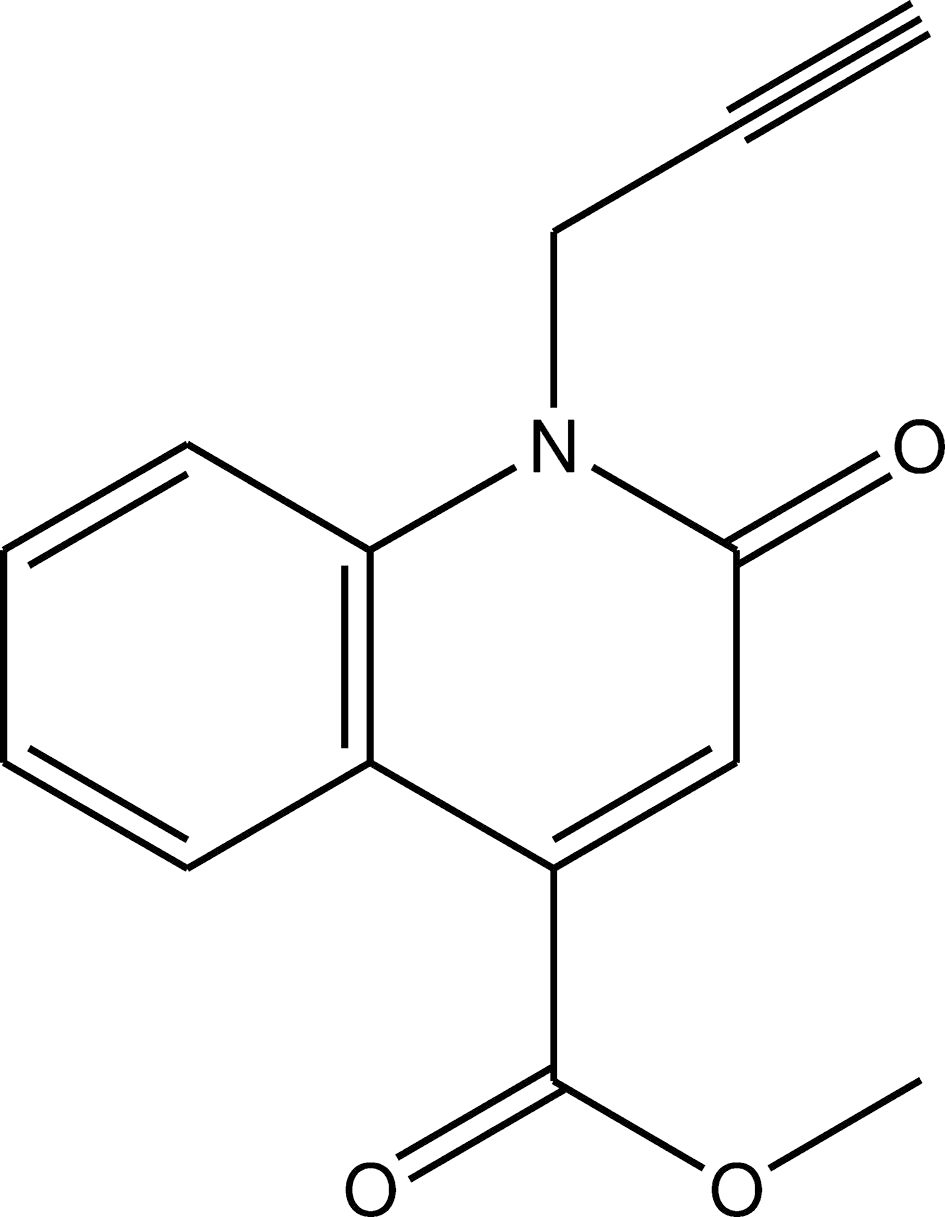


## 2. Structural commentary

**Figure 1:**
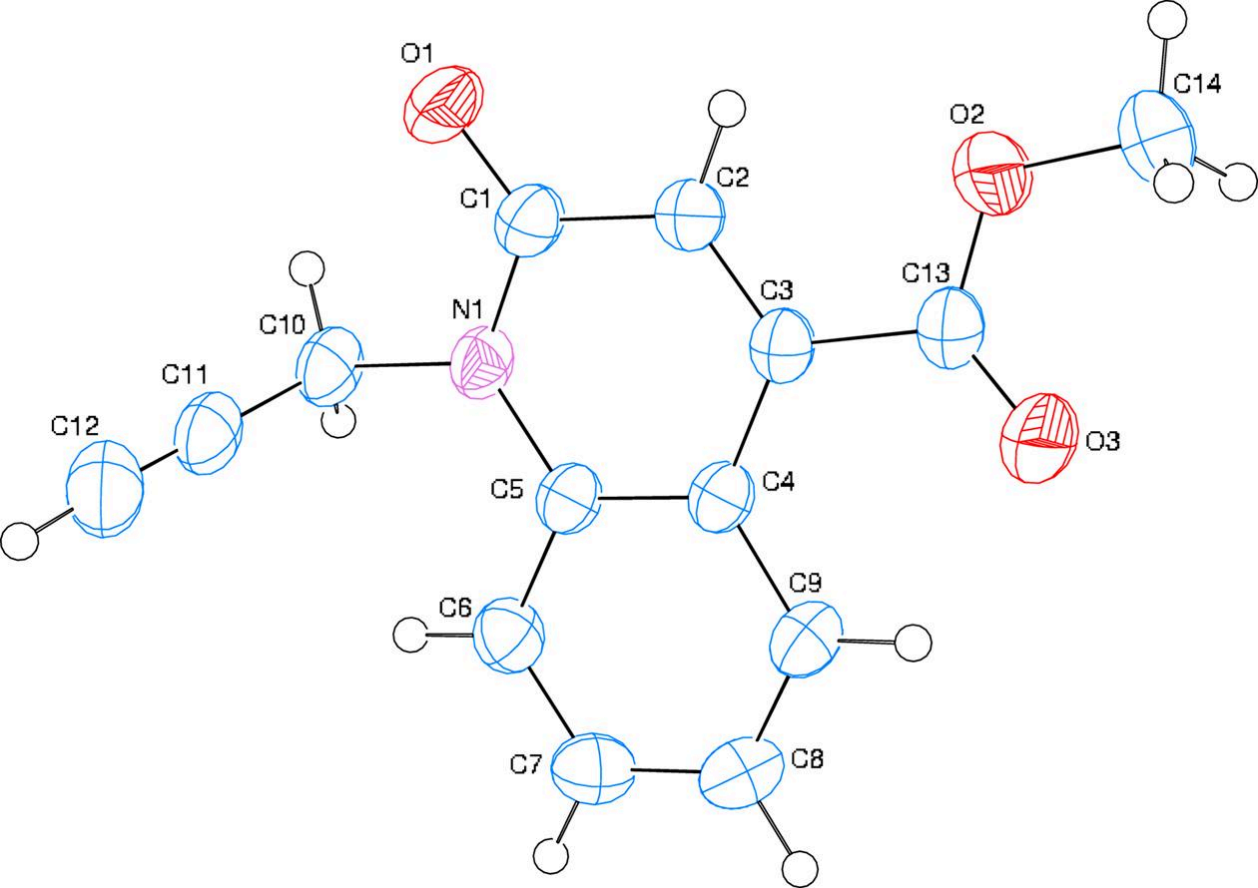
The mol­ecular structure of the title com­pound with the atom-labeling scheme and displacement ellipsoids drawn at the 50% probability level.

The di­hydro­quinoline core of the title mol­ecule (Fig. 1 ▸) deviates slightly from planarity, as indicated by the dihedral angle of 1.07 (3)° between the mean planes of the *A* (C1–C5/N1) and *B* (C4–C9) rings. Atoms O1, O2, O3, C10, C13 and C14 are −0.1294 (11), 0.1907 (12), −0.2708 (15), 0.0177 (14), −0.0267 (13) and 0.0953 (23) Å from the least-squares plane of the *A* ring. The O2—C13 [1.3123 (17) Å] and O3—C13 [1.1955 (16) Å] distances in the ester group indicate localized single and double bonds, rather than delocalized bonding arrangements. The O2—C13—O3 bond angle [122.55 (12)°] seems to be slightly increased with respect to that present in a free acid (122.2°; Sim *et al.*, 1955 ▸). The O2—C13—O3 bond angle may be com­pared with the corresponding value of 124.27 (17)° in di­aqua­bis­(2-bromo­benzoato-κ*O*)bis­(nicotinamide-κ*N* ^1^)zinc(II) (Hökelek *et al.*, 2009 ▸).

## 3. Supra­molecular features

**Figure 2:**
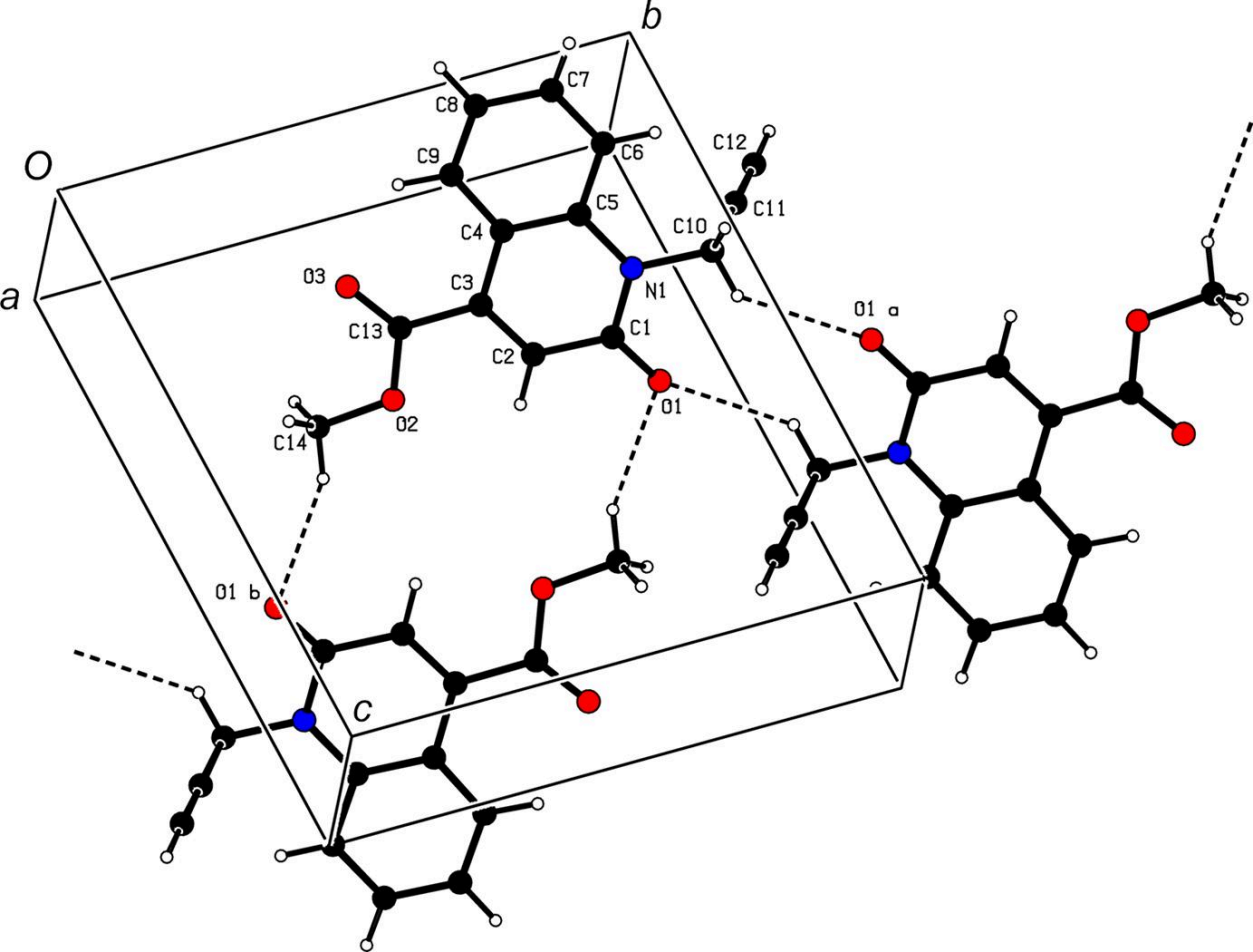
A partial packing diagram, viewed down the *a* axis, with C—H⋯O hydro­gen bonds shown as dashed lines.

In the crystal, C—H⋯O hydro­gen bonds (Table 1 ▸) link the mol­ecules, enclosing 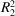 (10) and 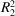 (16) ring motifs, into layers almost parallel to the *bc* plane (Fig. 2 ▸). These layers are further connected by π–π stacking inter­actions between the *A* and *B*(*x* − 1, *y*, *z*) rings [centroid-to-centroid distance = 3.5629 (7) Å, α = 1.13° and slippage = 1.221 Å] to form a triperiodic network.

## 4. Hirshfeld surface analysis

**Figure 3:**
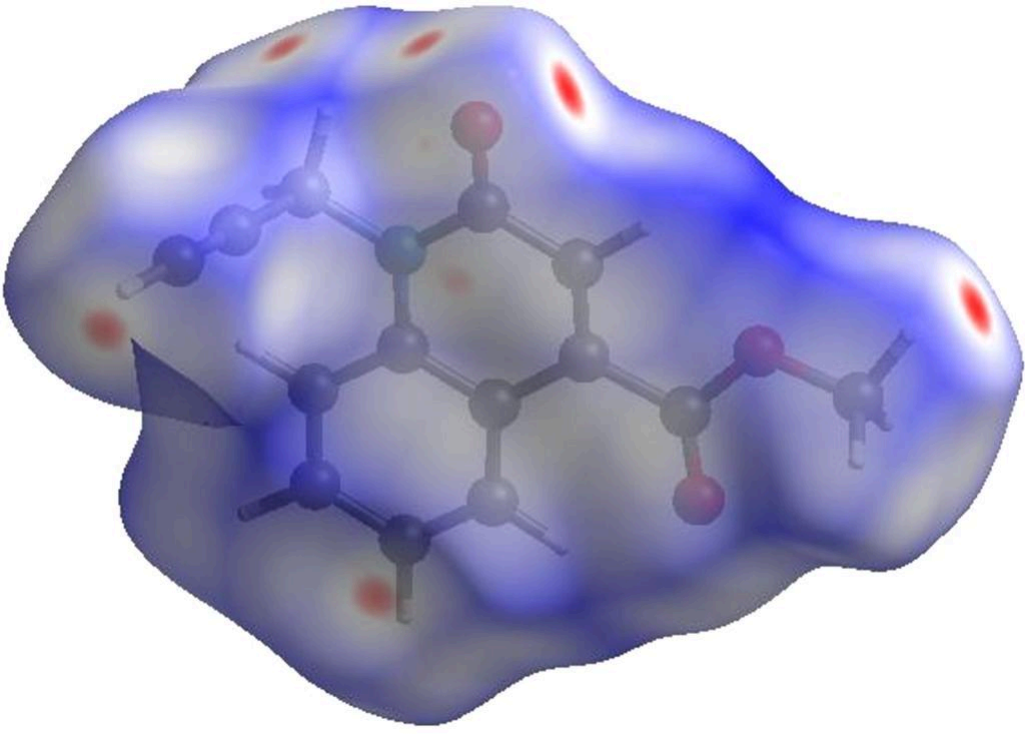
View of the three-dimensional Hirshfeld surface of the title com­pound, plotted over *d*_norm_ in the range from −0.1226 to 1.1991 a.u.

**Figure 4:**
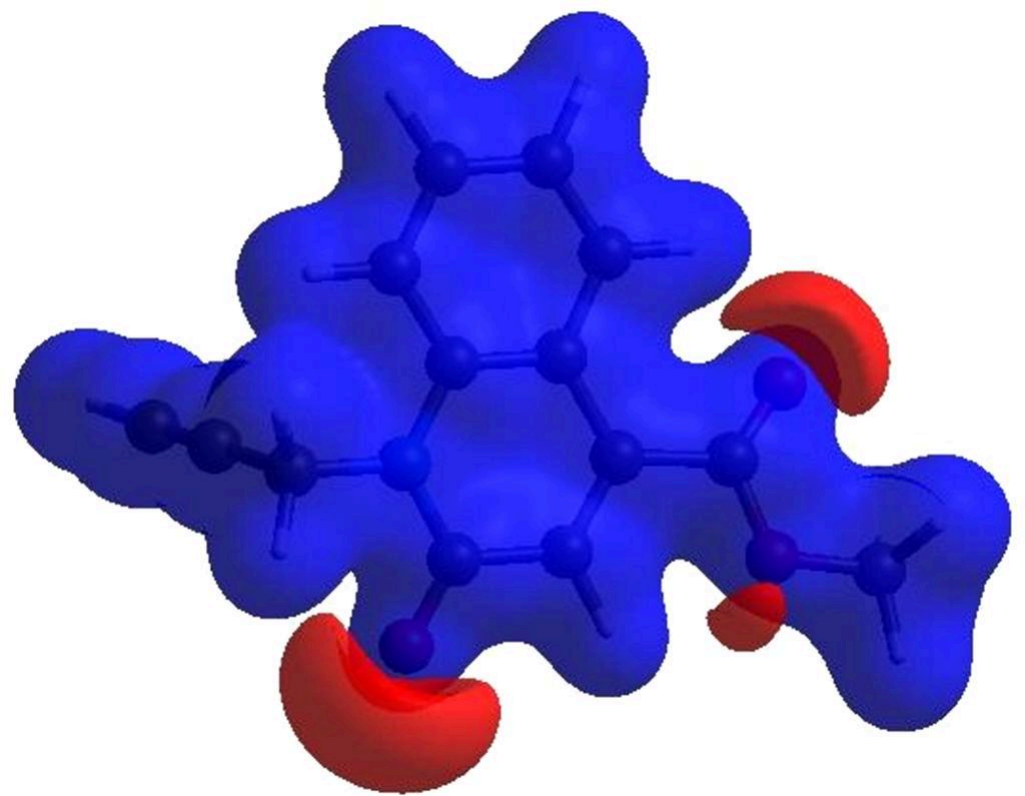
View of the three-dimensional Hirshfeld surface of the title com­pound plotted over electrostatic potential energy in the range from −0.0500 to 0.0500 a.u., using the STO-3G basis set at the Hartree–Fock level of theory.

**Figure 5:**
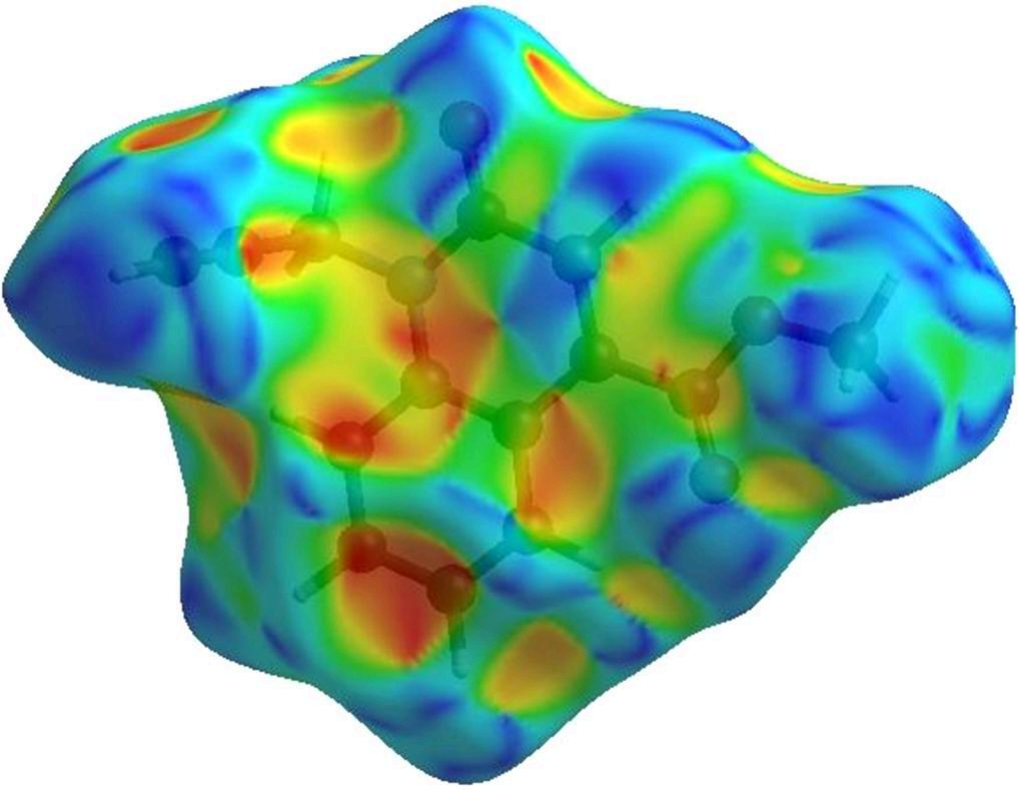
The Hirshfeld surface of the title com­pound plotted over shape-index.

**Figure 6:**
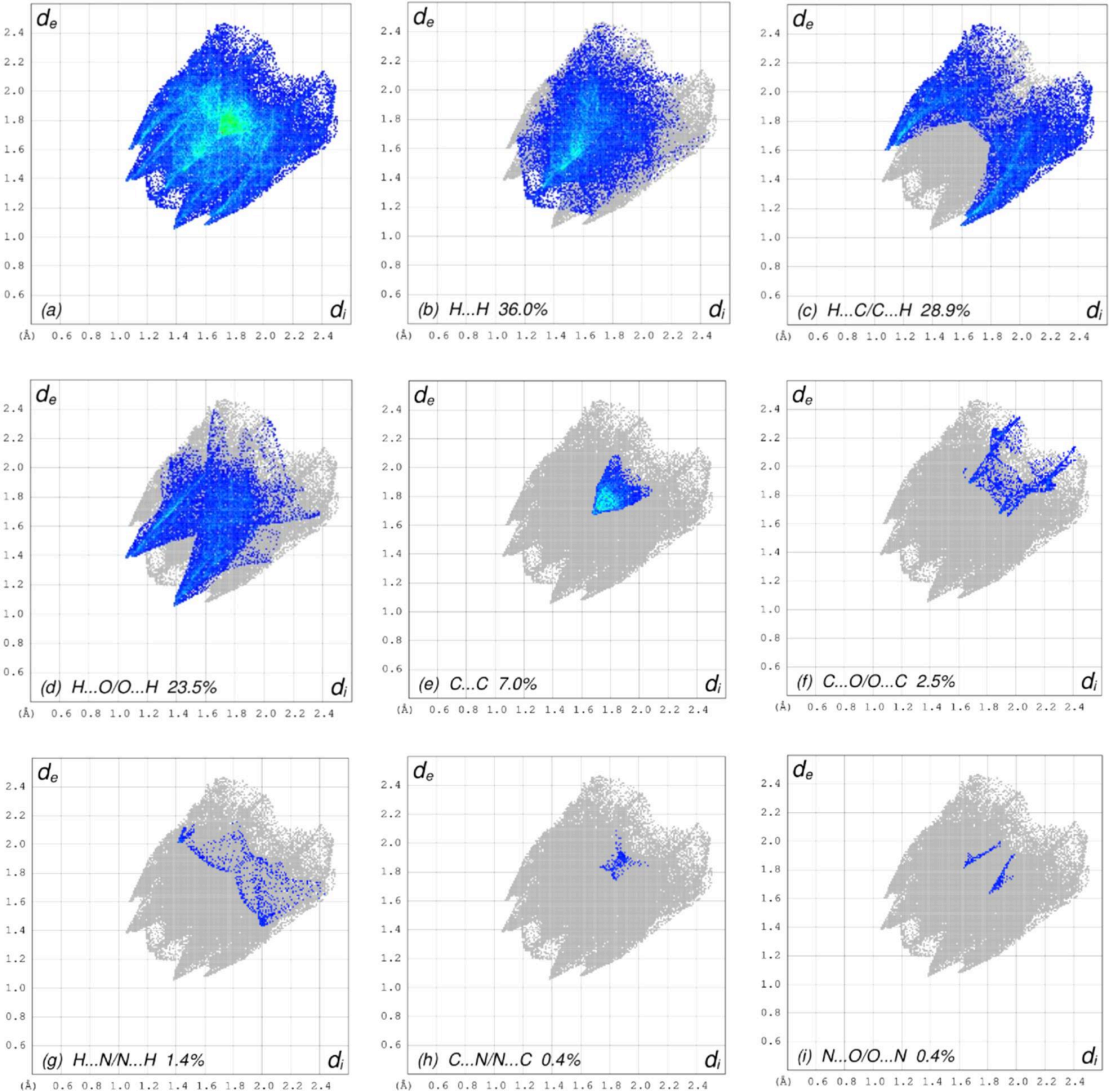
The full two-dimensional fingerprint plots for the title com­pound, showing (*a*) all inter­actions, (*b*) H⋯H, (*c*) H⋯C/C⋯H, (*d*) H⋯O/O⋯H, (*e*) C⋯C, (*f*) C⋯O/O⋯C, (*g*) H⋯N/N⋯H, (*h*) C⋯N/N⋯C and (*i*) N⋯O/O⋯N inter­actions. The *d*_i_ and *d*_e_ values are the closest inter­nal and external distances (in Å) from given points on the Hirshfeld surface contacts.

In order to visualize the inter­molecular inter­actions in the crystal of the title com­pound, a Hirshfeld surface (HS) analysis (Hirshfeld, 1977 ▸) was carried out by using *CrystalExplorer* (Spackman *et al.*, 2021 ▸). In the HS plotted over *d*_norm_ (Fig. 3 ▸), the white surface indicates contacts with distances equal to the sum of the van der Waals radii, and the red and blue colours indicate distances shorter (in close contact) or longer (distinct contact) than the sum of the van der Waals radii (Venkatesan *et al.*, 2016 ▸). The bright-red spots indicate their roles as respective donors and/or acceptors; they also appear as blue and red regions corresponding to positive and negative potentials on the HS mapped over electrostatic potential (Spackman *et al.*, 2008 ▸; Jayatilaka *et al.*, 2005 ▸), as shown in Fig. 4 ▸. The blue regions indicate positive electrostatic potential (hydro­gen-bond donors), while the red regions indicate negative electrostatic potential (hydro­gen-bond acceptors). The shape-index of the HS is a tool to visualize the π–π stacking inter­actions by the presence of adjacent red and blue triangles (Fig. 5 ▸). The overall two-dimensional fingerprint plot [Fig. 6 ▸(*a*)] and those delineated into H⋯H, H⋯C/C⋯H, H⋯O/O⋯H, C⋯C, C⋯O/O⋯C, H⋯N/N⋯H, C⋯N/N⋯C and N⋯O/O⋯N contacts (McKinnon *et al.*, 2007 ▸) are illustrated in Figs. 6 ▸(*b*)–(*i*), respectively, together with their relative contributions to the Hirshfeld surface. The most important inter­action is H⋯H, contributing 36.0% to the overall crystal packing, which is reflected in Fig. 6 ▸(*b*) as widely scattered points of high density due to the large hydro­gen content of the mol­ecule with the tip at *d*_e_ = *d*_i_ = 1.22 Å. In the absence of C—H⋯π inter­actions, the pair of characteristic wings resulting in the fingerprint plot delineated into H⋯C/C⋯H contacts [Fig. 6 ▸(*c*)] have a 28.9% contribution to the HS, with the tips at *d*_e_ + *d*_i_ = 2.68 Å. The pair of the scattered points of spikes resulting in the fingerprint plot delineated into H⋯O/O⋯H contacts [Fig. 6 ▸(*d*)], with a 23.5% contribution to the HS, has an almost symmetric distribution of points, with the tips at *d*_e_ + *d*_i_ = 2.44 Å. The C⋯C contacts [Fig. 6 ▸(*e*)] appear as an arrow-shaped distribution of points and have a contribution of 7.0% to the HS with the tip at *d*_e_ = *d*_i_ = 1.69 Å. The tiny spikes of C⋯O/O⋯C contacts [Fig. 6 ▸(*f*)], with a 2.5% contribution to the HS, are visible at *d*_e_ + *d*_i_ = 3.58 Å. Finally, the H⋯N/N⋯H [Fig. 6 ▸(*g*)], C⋯N/N⋯C [Fig. 6 ▸(*h*)] and N⋯O/O⋯N [Fig. 6 ▸(*i*)] contacts contribute 1.4, 0.4 and 0.4%, respectively, to the HS.

**Figure 7:**
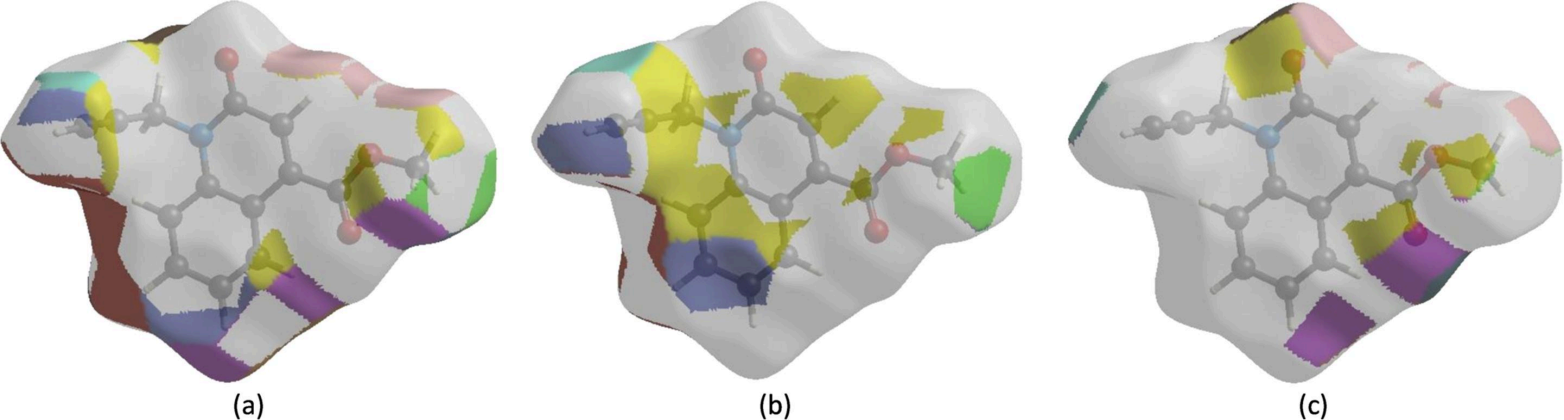
The Hirshfeld surface representations with the function *d*_norm_ plotted onto the surface for (*a*) H⋯H, (*b*) H⋯C/C⋯H and (*c*) H⋯O/O⋯H inter­actions.

The Hirshfeld surface representations with the function *d*_norm_ plotted onto the surface are shown for the H⋯H and H⋯C/C⋯H inter­actions in Figs. 7 ▸(*a*)–(*c*), respectively.

The Hirshfeld surface analysis confirms the importance of H-atom contacts in establishing the packing. The large number of H⋯H, H⋯C/C⋯H and H⋯O/O⋯H inter­actions suggest that van der Waals inter­actions play the major role in the crystal packing (Hathwar *et al.*, 2015 ▸).

## 5. Inter­action energy calculations and energy frameworks

**Figure 8:**
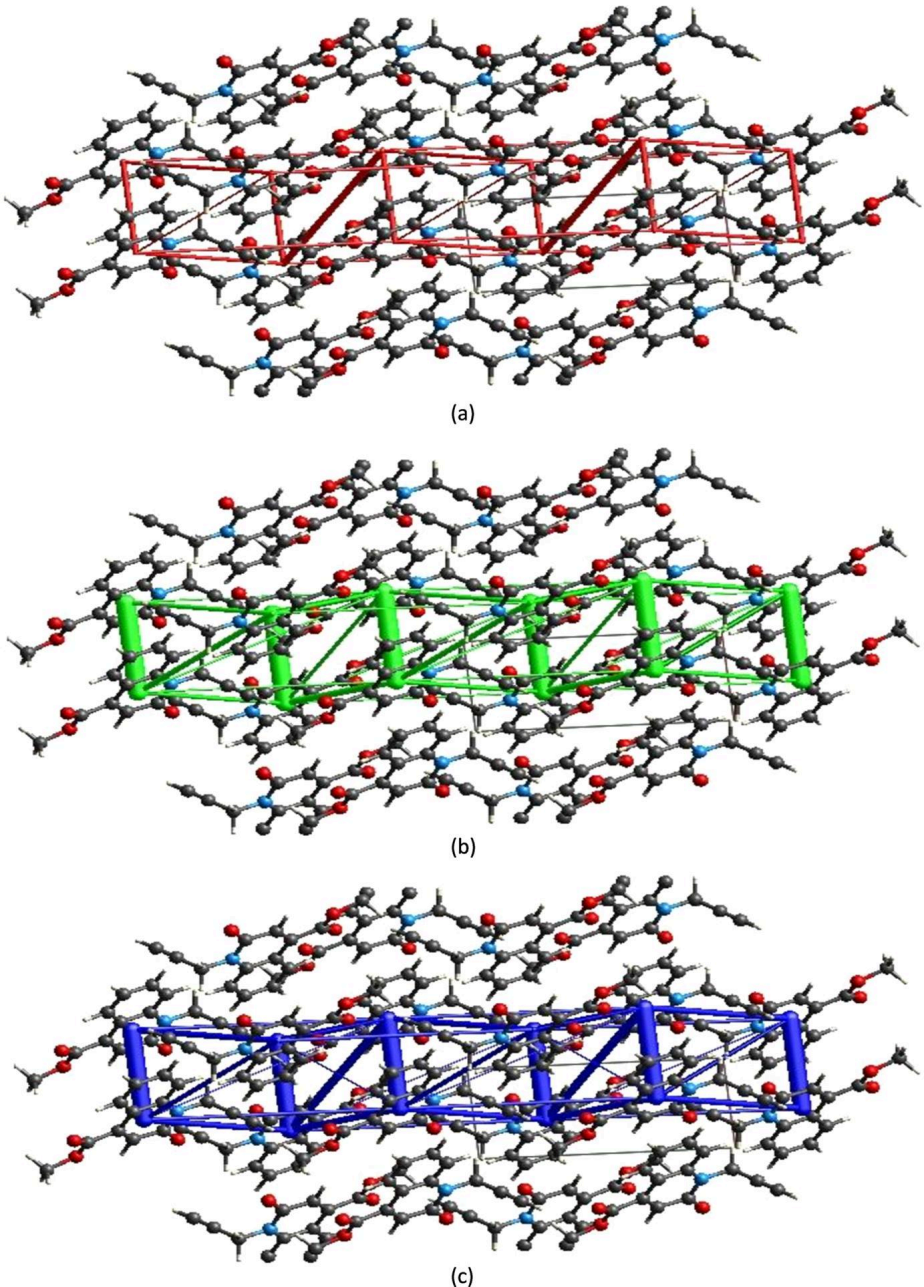
The energy frameworks, viewed down the *c* axis, for a cluster of mol­ecules of the title com­pound, showing the (*a*) electrostatic energy, (*b*) dispersion energy and (*c*) total energy diagrams, where the *b* axis is vertical and the *c* axis is horizontal. The cylindrical radius is proportional to the relative strength of the corresponding energies and was adjusted to the same scale factor of 80 with a cut-off value of 5 kJ mol^−1^ within 2 × 2 × 2 unit cells.

Using *CrystalExplorer* (Spackman *et al.*, 2021 ▸), the inter­molec­ular inter­action energies were calculated at the CEB3LYP/631G(d,p) energy level, where a cluster of mol­ecules is generated by applying crystallographic symmetry operations with respect to a selected central mol­ecule within a radius of 3.8 Å by default (Turner *et al.*, 2014 ▸). The total inter­molecular energy (*E*_tot_) is the sum of electrostatic (*E*_ele_), polarization (*E*_pol_), dispersion (*E*_dis_) and exchange–repulsion (*E*_rep_) energies (Turner *et al.*, 2015 ▸), with scale factors of 1.057, 0.740, 0.871 and 0.618, respectively (Mackenzie *et al.*, 2017 ▸). Energy frameworks combine the calculation of inter­molecular inter­action energies with a graphical representation of their magnitude (Turner *et al.*, 2015 ▸). Energies between mol­ecular pairs are represented as cylinders joining the centroids of pairs of mol­ecules with the cylinder radius proportional to the relative strength of the corresponding inter­action energy. Energy frameworks were constructed for *E*_ele_ (red cylinders), *E*_dis_ (green cylinders) and *E*_tot_ (blue cylinders), and are shown in Figs. 8 ▸(*a*)–(*c*). The evaluation of the electrostatic, dispersion and total energy frameworks indicates that in the title com­pound the stabilization is dominated by the dispersion energy contribution.

## 6. DFT calculations

**Figure 9:**
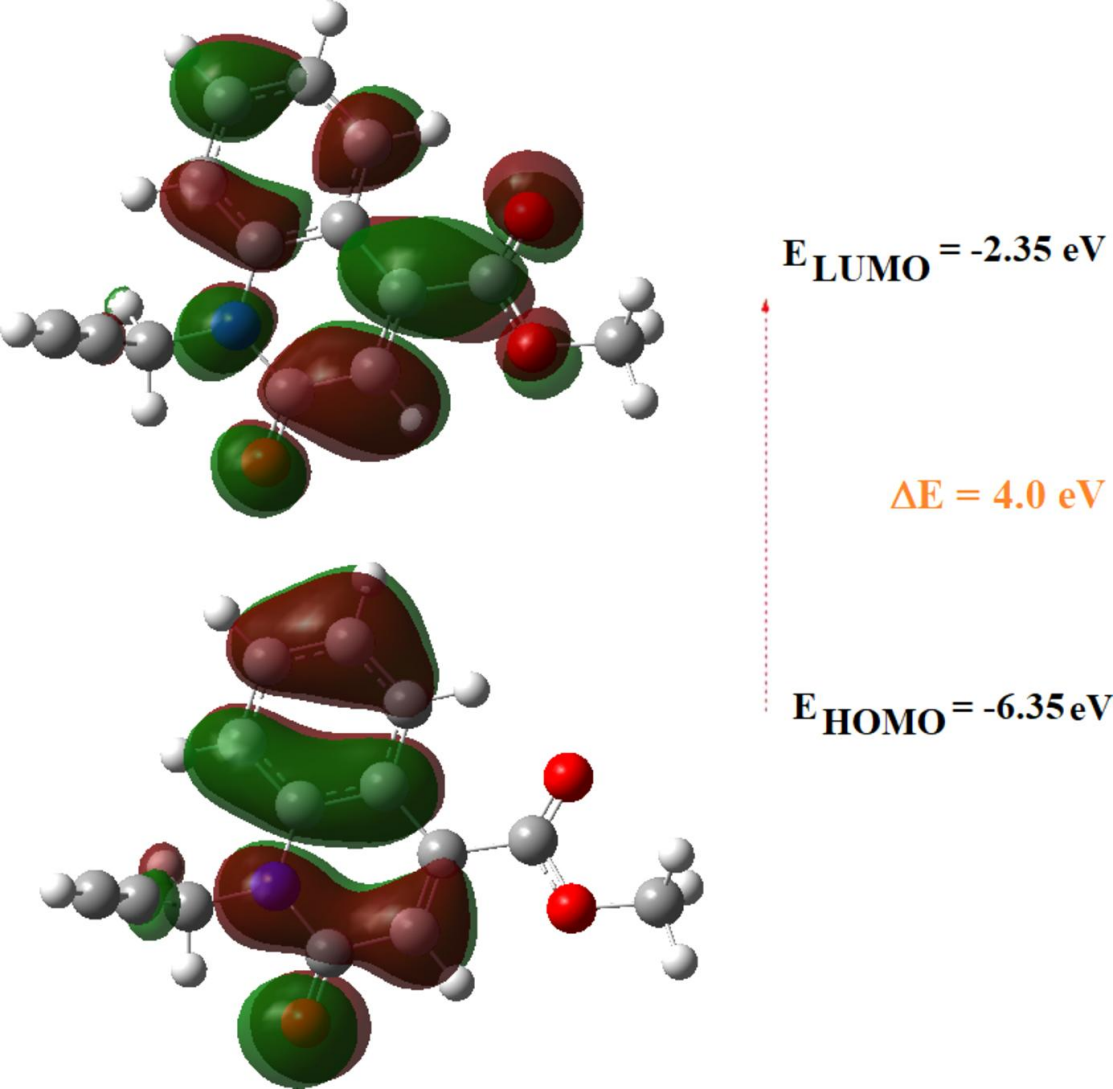
The energy band gap of the title com­pound.

The optimized structure of the title com­pound in the gas phase was com­puted on the basis of density functional theory (DFT) using the standard B3LYP functional and the 6311G(d,p) basis set (Becke, 1993 ▸), as implemented in *GAUSSIAN09* (Frisch *et al.*, 2009 ▸). Comparisons of calculated bond lengths and angles with those of the experimental study are com­piled in Table 2 ▸. The frontier orbitals were also investigated, and the highest occupied mol­ecular orbital (HOMO) and lowest unoccupied mol­ecular orbital (LUMO) orbitals are depicted in Fig. 9 ▸. It can be seen that the electron density of the HOMO is mostly distributed within the quinoline moiety, while that of the LUMO is mostly distributed over the carboxylate group.

Other chemistry descriptors (chemical hardness η, softness *S*, electronegativity χ and electrophilicity ω) derived from the conceptual DFT calculations are given in Table 3 ▸. The HOMO and LUMO are localized in the plane extending from the methyl 2-oxo-1-(prop-2-yn­yl)-1,2-di­hydro­quinoline-4-car­box­yl­­ate ring. The energy band gap [Δ*E* = *E*_LUMO_ − *E*_HOMO_] (Fig. 9 ▸) of the mol­ecule is about −4.0 eV, with individual frontier mol­ecular orbital energies, *E*_HOMO_ and *E*_LUMO_, of −6.35 and −2.35 eV, respectively.

## 7. Database survey

**Figure 10:**
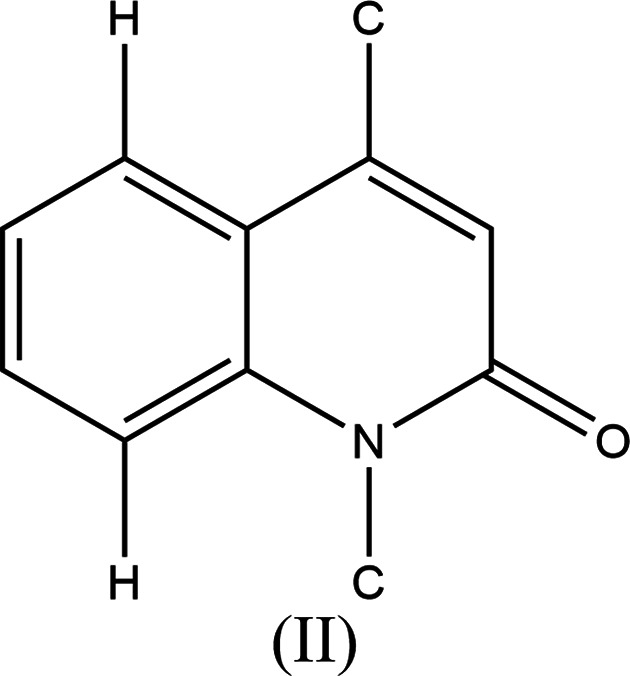
The mol­ecular moiety used for the database search.

A search of the Cambridge Structural Database (CSD, updated 20 March 2023; Groom *et al.*, 2016 ▸) using fragment (II) (Fig. 10 ▸) returned 20 hits, 16 of which contained an ester group attached to C7 (the rest contained an alkyl group at this position) and, only two of them, with refcodes ROKCIG (Filali Baba *et al.*, 2019 ▸) and REYREV (Filali Baba *et al.*, 2017 ▸), contain halogen atoms attached to aromatic rings. The former is more closely related to the title mol­ecule due to the presence of an ethyl group on the nitro­gen and ester substituents. Unlike the title mol­ecule, that of ROKCIG forms an inverted dimer *via* C—H⋯O hydro­gen bonds (instead of ribbons), with layer-by-layer connections approximately parallel to (10 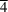 ), but it has no C—H⋯Cl hydro­gen bonds or π–π stacking inter­actions. The halogen-free analogue of ROKCIG (ROKCOM; Filali Baba *et al.*, 2019 ▸) uses C—H⋯O hydro­gen bonds to form mol­ecular bands along the *c* axis, which are connected by weak π–π inter­actions.

## 8. Refinement

Crystal, data collection and refinement details are presented in Table 4 ▸. H atoms were included as riding contributions in idealized positions with isotropic displacement parameters tied to those of the attached atoms. Two reflections obscured by the beamstop were omitted from the final refinement.

## 9. Synthesis and crystallization

To a solution of methyl 2-oxo-1,2-di­hydro­quinoline-4-car­box­yl­ate (4.47 mmol) in 10 ml of di­methyl­formamide (DMF) were added propargyl bromide (9.83 mmol), K_2_CO_3_ (22.36 mmol) and tetra-*n*-butyl­ammonium bromide (TBAB; 0.5 mmol). The reaction mixture was stirred at room tem­per­a­ture in DMF for 6 h. After removal of the formed salts, the solvent was evaporated under reduced pressure and the residue obtained was dissolved in di­chloro­methane. The organic phase was dried over Na_2_SO_4_ and then concentrated *in vacuo*. A pure com­pound was obtained after recrystallization from di­chloro­methane/hexane (2:3 *v*/*v*).

## Supplementary Material

Crystal structure: contains datablock(s) I, global. DOI: 10.1107/S2056989023007557/wm5688sup1.cif

Structure factors: contains datablock(s) I. DOI: 10.1107/S2056989023007557/wm5688Isup2.hkl

Supporting information file. DOI: 10.1107/S2056989023007557/wm5688Isup3.cdx

Supporting information file. DOI: 10.1107/S2056989023007557/wm5688Isup4.cdx

CCDC reference: 2291603

Additional supporting information:  crystallographic information; 3D view; checkCIF report

## Figures and Tables

**Table 1:** Hydro­gen-bond geometry (Å, °)

*D*—H⋯*A*	*D*—H	H⋯*A*	*D*⋯*A*	*D*—H⋯*A*
C9—H9⋯O3	0.969 (17)	2.210 (15)	2.8807 (19)	125.3 (12)
C10—H10*A*⋯O1	0.922 (16)	2.277 (17)	2.6961 (17)	107.1 (12)
C14—H14*B*⋯O1^i^	0.95 (2)	2.56 (2)	3.433 (2)	153.8 (18)

Symmetry code: (i) 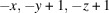 .

**Table 2:** Comparison (X-ray and DFT) of selected bond lengths and angles (Å, °)

Bonds/angles	X-ray	B3LYP/6-311G(d,p)
O1—C1	1.2288 (14)	1.2231
N1—C5	1.3993 (14)	1.3955
N1—C10	1.4742 (14)	1.4727
N1—C1	1.3774 (16)	1.4035
O2—C13	1.3123 (17)	1.3460
O2—C14	1.4491 (17)	1.4399
O3—C13	1.1955 (16)	1.2081
C5—C4	1.4159 (16)	1.4234
C5—C6	1.4011 (17)	1.4062
C4—C3	1.4516 (16)	1.4539
C4—C9	1.4064 (16)	1.4096
		
C5—N1—C10	120.18 (10)	120.925
C1—N1—C5	123.16 (9)	123.436
C1—N1—C10	116.52 (10)	115.623
C13—O2—C14	116.39 (12)	115.680
N1—C5—C4	120.23 (10)	120.142
N1—C5—C6	119.97 (10)	120.504
C6—C5—C4	119.80 (10)	119.355
C5—C4—C3	117.38 (10)	117.701
C9—C4—C5	118.06 (11)	118.477

**Table 3:** Calculated energies for com­pound (I)

Total energy, TE (eV)	−22331.1678
*E*_HOMO_ (eV)	–6,35
*E*_LUMO_ (eV)	–2.35
Gap, Δ*E* (eV)	–4.0
Dipole moment, μ (Debye)	2.1062
Ionization potential, *I* (eV)	6.35
Electron affinity, *A*	2.35
Electronegativity, χ	4.35
Hardness, η	2
Electrophilicity index, ω	4.73
Softness, σ	0.5
Fraction of electron transferred, Δ*N*	0.66

**Table 4:** Experimental details

Crystal data	
Chemical formula	C_14_H_11_NO_3_
*M*_r_	241.24
Crystal system, space group	Triclinic, *P* 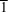
Temperature (K)	296
*a*, *b*, *c* (Å)	4.7033 (2), 11.1113 (6), 11.3876 (5)
α, β, γ (°)	81.759 (2), 83.356 (2), 85.564 (2)
*V* (Å^3^)	583.89 (5)
*Z*	2
Radiation type	Mo *K*α
μ (mm^−1^)	0.10
Crystal size (mm)	0.24 × 0.14 × 0.11

Data collection	
Diffractometer	Bruker DUO PHOTON III
Absorption correction	Multi-scan (*SADABS*; Krause *et al.*, 2015 ▸)
*T*_min_, *T*_max_	0.708, 0.746
No. of measured, independent and observed [*I* > 2σ(*I*)] reflections	45341, 3559, 2489
*R*_int_	0.045
(sin θ/λ)_max_ (Å^−1^)	0.714

Refinement	
*R*[*F* ^2^ > 2σ(*F* ^2^)], *wR*(*F* ^2^), *S*	0.047, 0.148, 1.11
No. of reflections	3559
No. of parameters	207
H-atom treatment	All H-atom parameters refined
Δρ_max_, Δρ_min_ (e Å^−3^)	0.38, −0.28

Computer programs: *APEX3* and *SAINT* (Bruker, 2019 ▸), *SHELXT* (Sheldrick, 2015*a* ▸), *SHELXL* (Sheldrick, 2015*b* ▸) and *OLEX2* (Dolomanov *et al.*, 2009 ▸).

## supplementary crystallographic information

### Crystal data

**Table:**

C_14_H_11_NO_3_	*Z* = 2
*M**_r_* = 241.24	*F*(000) = 252
Triclinic, *P*1	*D*_x_ = 1.372 Mg m^−^^3^
*a* = 4.7033 (2) Å	Mo *K*α radiation, λ = 0.71073 Å
*b* = 11.1113 (6) Å	Cell parameters from 9957 reflections
*c* = 11.3876 (5) Å	θ = 2.4–30.2°
α = 81.759 (2)°	µ = 0.10 mm^−^^1^
β = 83.356 (2)°	*T* = 296 K
γ = 85.564 (2)°	Block, colourless
*V* = 583.89 (5) Å^3^	0.24 × 0.14 × 0.11 mm

### Data collection

**Table:**

Bruker DUO PHOTON III diffractometer	*R*_int_ = 0.045
Radiation source: microfocus sealed X-ray tube	θ_max_ = 30.5°, θ_min_ = 1.8°
φ and ω scans	*h* = −6→6
Absorption correction: multi-scan (SADABS; Krause *et al.*, 2015)	*k* = −15→15
*T*_min_ = 0.708, *T*_max_ = 0.746	*l* = −16→16
45341 measured reflections	3 standard reflections every 1000 reflections
3559 independent reflections	intensity decay: 1%
2489 reflections with *I* > 2σ(*I*)	

### Refinement

**Table:**

Refinement on *F*^2^	Primary atom site location: dual
Least-squares matrix: full	Secondary atom site location: difference Fourier map
*R*[*F*^2^ > 2σ(*F*^2^)] = 0.047	Hydrogen site location: difference Fourier map
*wR*(*F*^2^) = 0.148	All H-atom parameters refined
*S* = 1.11	*w* = 1/[σ^2^(*F*_o_^2^) + (0.0712*P*)^2^ + 0.064*P*] where *P* = (*F*_o_^2^ + 2*F*_c_^2^)/3
3559 reflections	(Δ/σ)_max_ < 0.001
207 parameters	Δρ_max_ = 0.38 e Å^−^^3^
0 restraints	Δρ_min_ = −0.28 e Å^−^^3^

### Special details

**Table:**

Geometry. All esds (except the esd in the dihedral angle between two l.s. planes) are estimated using the full covariance matrix. The cell esds are taken into account individually in the estimation of esds in distances, angles and torsion angles; correlations between esds in cell parameters are only used when they are defined by crystal symmetry. An approximate (isotropic) treatment of cell esds is used for estimating esds involving l.s. planes.

### Fractional atomic coordinates and isotropic or equivalent isotropic displacement parameters (Å^2^)

**Table:**

	*x*	*y*	*z*	*U*_iso_*/*U*_eq_	
O1	0.3589 (2)	0.16022 (9)	0.53718 (9)	0.0571 (3)	
N1	0.6716 (2)	0.14916 (9)	0.67741 (9)	0.0381 (2)	
O2	0.2219 (3)	0.56421 (9)	0.64710 (10)	0.0655 (3)	
O3	0.3962 (3)	0.56270 (10)	0.81846 (12)	0.0812 (4)	
C5	0.7779 (2)	0.19819 (10)	0.76892 (10)	0.0359 (2)	
C4	0.6875 (2)	0.31800 (10)	0.79156 (10)	0.0357 (2)	
C3	0.4848 (3)	0.38599 (10)	0.71625 (10)	0.0372 (3)	
C10	0.7636 (3)	0.02397 (11)	0.65470 (13)	0.0441 (3)	
C13	0.3695 (3)	0.51328 (11)	0.73414 (12)	0.0434 (3)	
C11	0.6261 (3)	−0.06721 (11)	0.74351 (13)	0.0463 (3)	
C6	0.9724 (3)	0.12821 (12)	0.83921 (12)	0.0439 (3)	
C1	0.4663 (3)	0.20952 (11)	0.60989 (11)	0.0411 (3)	
C2	0.3857 (3)	0.33419 (11)	0.63055 (11)	0.0422 (3)	
C9	0.8005 (3)	0.36311 (12)	0.88452 (12)	0.0438 (3)	
C8	0.9935 (3)	0.29352 (14)	0.95173 (12)	0.0500 (3)	
C7	1.0789 (3)	0.17610 (14)	0.92919 (13)	0.0497 (3)	
C12	0.5140 (4)	−0.14126 (14)	0.81495 (17)	0.0605 (4)	
C14	0.0863 (5)	0.68407 (15)	0.6591 (2)	0.0686 (5)	
H10A	0.720 (4)	0.0174 (14)	0.5791 (15)	0.053 (4)*	
H9	0.736 (3)	0.4439 (15)	0.9029 (14)	0.054 (4)*	
H10B	0.974 (3)	0.0129 (13)	0.6502 (12)	0.044 (4)*	
H6	1.030 (3)	0.0475 (15)	0.8245 (14)	0.052 (4)*	
H2	0.247 (4)	0.3772 (15)	0.5795 (15)	0.056 (4)*	
H8	1.067 (4)	0.3254 (16)	1.0161 (17)	0.070 (5)*	
H7	1.214 (4)	0.1274 (17)	0.9743 (16)	0.066 (5)*	
H14A	0.232 (6)	0.741 (2)	0.657 (2)	0.111 (8)*	
H14B	−0.011 (5)	0.7061 (19)	0.590 (2)	0.083 (6)*	
H14C	−0.036 (6)	0.684 (2)	0.729 (2)	0.100 (8)*	
H12	0.435 (5)	−0.201 (2)	0.874 (2)	0.091 (7)*	

### Atomic displacement parameters (Å^2^)

**Table:**

	*U*^11^	*U*^22^	*U*^33^	*U*^12^	*U*^13^	*U*^23^
O1	0.0699 (7)	0.0505 (6)	0.0589 (6)	0.0021 (5)	−0.0237 (5)	−0.0248 (5)
N1	0.0416 (5)	0.0328 (5)	0.0416 (5)	0.0006 (4)	−0.0036 (4)	−0.0131 (4)
O2	0.0899 (8)	0.0451 (6)	0.0658 (7)	0.0234 (5)	−0.0303 (6)	−0.0185 (5)
O3	0.1245 (11)	0.0485 (6)	0.0815 (8)	0.0261 (6)	−0.0469 (8)	−0.0339 (6)
C5	0.0346 (5)	0.0359 (6)	0.0379 (6)	−0.0031 (4)	−0.0010 (4)	−0.0092 (4)
C4	0.0357 (5)	0.0355 (6)	0.0367 (5)	−0.0027 (4)	−0.0009 (4)	−0.0097 (4)
C3	0.0401 (6)	0.0323 (5)	0.0395 (6)	−0.0015 (4)	−0.0015 (4)	−0.0083 (4)
C10	0.0468 (7)	0.0369 (6)	0.0508 (7)	0.0033 (5)	−0.0026 (5)	−0.0189 (5)
C13	0.0488 (7)	0.0337 (6)	0.0488 (7)	−0.0004 (5)	−0.0053 (5)	−0.0097 (5)
C11	0.0466 (7)	0.0352 (6)	0.0607 (8)	0.0035 (5)	−0.0097 (6)	−0.0185 (6)
C6	0.0423 (6)	0.0402 (6)	0.0491 (7)	0.0027 (5)	−0.0061 (5)	−0.0075 (5)
C1	0.0461 (6)	0.0387 (6)	0.0407 (6)	−0.0015 (5)	−0.0056 (5)	−0.0125 (5)
C2	0.0485 (7)	0.0368 (6)	0.0425 (6)	0.0022 (5)	−0.0101 (5)	−0.0085 (5)
C9	0.0460 (6)	0.0440 (7)	0.0447 (6)	−0.0034 (5)	−0.0051 (5)	−0.0161 (5)
C8	0.0501 (7)	0.0602 (8)	0.0438 (7)	−0.0047 (6)	−0.0105 (6)	−0.0154 (6)
C7	0.0454 (7)	0.0572 (8)	0.0472 (7)	0.0010 (6)	−0.0119 (6)	−0.0055 (6)
C12	0.0642 (9)	0.0447 (8)	0.0737 (10)	−0.0063 (7)	−0.0086 (8)	−0.0093 (7)
C14	0.0861 (13)	0.0412 (8)	0.0810 (12)	0.0209 (8)	−0.0280 (11)	−0.0148 (8)

### Geometric parameters (Å, º)

**Table:**

O1—C1	1.2288 (14)	C10—H10B	0.983 (15)
N1—C5	1.3993 (14)	C11—C12	1.180 (2)
N1—C10	1.4742 (14)	C6—C7	1.3785 (19)
N1—C1	1.3774 (16)	C6—H6	0.949 (17)
O2—C13	1.3123 (17)	C1—C2	1.4524 (17)
O2—C14	1.4491 (17)	C2—H2	0.977 (18)
O3—C13	1.1955 (16)	C9—C8	1.3750 (19)
C5—C4	1.4159 (16)	C9—H9	0.969 (17)
C5—C6	1.4011 (17)	C8—C7	1.386 (2)
C4—C3	1.4516 (16)	C8—H8	0.966 (19)
C4—C9	1.4064 (16)	C7—H7	0.950 (19)
C3—C13	1.5081 (16)	C12—H12	0.94 (2)
C3—C2	1.3449 (17)	C14—H14A	0.97 (3)
C10—C11	1.457 (2)	C14—H14B	0.95 (2)
C10—H10A	0.922 (16)	C14—H14C	0.93 (3)
			
C5—N1—C10	120.18 (10)	C7—C6—C5	120.28 (12)
C1—N1—C5	123.16 (9)	C7—C6—H6	120.2 (9)
C1—N1—C10	116.52 (10)	O1—C1—N1	121.71 (11)
C13—O2—C14	116.39 (12)	O1—C1—C2	122.56 (12)
N1—C5—C4	120.23 (10)	N1—C1—C2	115.73 (10)
N1—C5—C6	119.97 (10)	C3—C2—C1	123.09 (11)
C6—C5—C4	119.80 (10)	C3—C2—H2	121.9 (10)
C5—C4—C3	117.38 (10)	C1—C2—H2	114.9 (10)
C9—C4—C5	118.06 (11)	C4—C9—H9	119.0 (10)
C9—C4—C3	124.56 (11)	C8—C9—C4	121.30 (12)
C4—C3—C13	121.83 (10)	C8—C9—H9	119.6 (10)
C2—C3—C4	120.15 (10)	C9—C8—C7	120.08 (12)
C2—C3—C13	117.97 (11)	C9—C8—H8	120.2 (11)
N1—C10—H10A	106.8 (10)	C7—C8—H8	119.7 (11)
N1—C10—H10B	109.5 (8)	C6—C7—C8	120.47 (13)
C11—C10—N1	112.13 (10)	C6—C7—H7	118.6 (11)
C11—C10—H10A	111.3 (10)	C8—C7—H7	120.9 (11)
C11—C10—H10B	111.7 (8)	C11—C12—H12	176.4 (14)
H10A—C10—H10B	105.1 (13)	O2—C14—H14A	109.5 (16)
O2—C13—C3	111.85 (10)	O2—C14—H14B	105.1 (13)
O3—C13—O2	122.55 (12)	O2—C14—H14C	111.5 (16)
O3—C13—C3	125.54 (12)	H14A—C14—H14B	107.6 (19)
C12—C11—C10	179.65 (16)	H14A—C14—H14C	110 (2)
C5—C6—H6	119.5 (9)	H14B—C14—H14C	113 (2)
			
O1—C1—C2—C3	174.62 (13)	C10—N1—C5—C4	−179.38 (10)
N1—C5—C4—C3	−0.33 (16)	C10—N1—C5—C6	0.02 (17)
N1—C5—C4—C9	179.99 (10)	C10—N1—C1—O1	2.22 (18)
N1—C5—C6—C7	179.74 (11)	C10—N1—C1—C2	−177.92 (10)
N1—C1—C2—C3	−5.23 (19)	C13—C3—C2—C1	−175.93 (11)
C5—N1—C10—C11	75.59 (14)	C6—C5—C4—C3	−179.73 (10)
C5—N1—C1—O1	−173.59 (11)	C6—C5—C4—C9	0.59 (17)
C5—N1—C1—C2	6.27 (18)	C1—N1—C5—C4	−3.71 (17)
C5—C4—C3—C13	178.69 (10)	C1—N1—C5—C6	175.68 (11)
C5—C4—C3—C2	1.31 (17)	C1—N1—C10—C11	−100.36 (13)
C5—C4—C9—C8	0.10 (19)	C2—C3—C13—O2	−11.40 (17)
C5—C6—C7—C8	0.4 (2)	C2—C3—C13—O3	165.96 (15)
C4—C5—C6—C7	−0.86 (19)	C9—C4—C3—C13	−1.66 (18)
C4—C3—C13—O2	171.16 (11)	C9—C4—C3—C2	−179.04 (12)
C4—C3—C13—O3	−11.5 (2)	C9—C8—C7—C6	0.3 (2)
C4—C3—C2—C1	1.54 (19)	C14—O2—C13—O3	−1.1 (2)
C4—C9—C8—C7	−0.5 (2)	C14—O2—C13—C3	176.33 (15)
C3—C4—C9—C8	−179.56 (12)		

### Hydrogen-bond geometry (Å, º)

**Table:**

*D*—H···*A*	*D*—H	H···*A*	*D*···*A*	*D*—H···*A*
C9—H9···O3	0.969 (17)	2.210 (15)	2.8807 (19)	125.3 (12)
C10—H10*A*···O1	0.922 (16)	2.277 (17)	2.6961 (17)	107.1 (12)
C14—H14*B*···O1^i^	0.95 (2)	2.56 (2)	3.433 (2)	153.8 (18)

Symmetry code: (i) −*x*, −*y*+1, −*z*+1.
